# Living on the edge of the community: factors associated with discontinuation of community living among people with cognitive impairment

**DOI:** 10.1186/s12877-021-02084-2

**Published:** 2021-02-19

**Authors:** Chiaki Ura, Tsuyoshi Okamura, Mika Sugiyama, Fumiko Miyamae, Mari Yamashita, Riko Nakayama, Ayako Edahiro, Tsutomu Taga, Hiroki Inagaki, Madoka Ogawa, Shuichi Awata

**Affiliations:** 1 Research Team for Promoting Independence and Mental Health, Tokyo Metropolitan Institute of Gerontology, 35-2, Sakae-cho, Itabashi-ku, Tokyo, 173-0015 Japan; 2 Research Team for Social Participation and Community Health, Tokyo Metropolitan Institute of Gerontology, Tokyo, Japan; 3 Department of Integrated Education and Science, Graduate School of Education, The University of Tokyo, Tokyo, Japan

**Keywords:** Cognitive impairment, Community-dwelling elderly, Discontinuation of community living, Housing

## Abstract

**Background:**

As Japanese society continues to age, the isolation of older people is increasing, and community living for people with cognitive impairment is becoming more difficult. However, the challenges faced by people with cognitive impairment living in the community have not been fully explored because of methodological difficulties. This study re-accessed people with cognitive impairment identified in a previous epidemiological survey to explore their current situation and the risk factors associated with all-cause discontinuation of community living.

**Methods:**

Under a community-based participatory framework, we examined a high-risk approach for people with cognitive impairment and a community action approach in parallel, to build a dementia-friendly community. For the high-risk approach, we achieved stepwise access to 7614 older residents, which enabled us to select and visit the homes of 198 participants with a Mini-Mental State Examination score < 24 in 2016. In 2019, we re-accessed these individuals. For the community action approach, we built a community space in the study area to build partnerships with community residents and community workers and were able to re-access participants using multiple methods.

**Results:**

We found that 126 (63.6%) participants had continued living in the same community, but 58 (29.3%) had discontinued community living. Of these, 18 (9.1%) had died, 18 (9.1%) were institutionalized, 9 (4.5%) were hospitalized, and 13 (6.6%) had moved out of the community. A multiple logistic regression analysis identified the following risk factors associated with discontinuation of community living: being certified under long-term care insurance, needing housing support, and needing rights protection.

**Conclusions:**

Three years after the baseline survey, 29.3% of people with cognitive impairment had discontinued community living. Despite having cognitive impairment or living alone, older people were able to continue living in the community if their needs for housing support and rights protection were met. Both social interventions and medical interventions are important to build age-friendly communities.

**Trial registration:**

UMIN, UMIN000038189, Registered 3 October 2019, https://upload.umin.ac.jp/cgi-open-bin/ctr_e/ctr_view.cgi?recptno=R000043521

**Supplementary Information:**

The online version contains supplementary material available at 10.1186/s12877-021-02084-2.

## Background

Japanese society is rapidly aging and there is a corresponding increase in the number of people with cognitive impairment [1]. A study that administered the Mini-Mental State Examination (MMSE) to all older residents living in one district of Tokyo (*n* = 1319) found that the ratio of those who scored below the cutoff criterion (i.e., 23/24) increased with age (6.0, 13.4, and 33.3% in individuals aged 65–74, 75–84, and ≥ 85 years, respectively) [2]. Building age-friendly and dementia-friendly communities is becoming a common goal for people with dementia, families, policymakers, and researchers [3]. The Japanese government has adopted an aging-in-place policy (the New Orange Plan) to improve the living environments of people with dementia by enabling them to continue living in familiar spaces and environments for as long as possible [4]. This is parallel to global trends, such as the National Health Services and Community Care Act 1990 in the United Kingdom, which prioritized providing care to individuals in their existing homes [5]. This philosophy was succeeded by the Care Act 2014 [6], which more strongly prioritized independent living, such as individuals’ control over their day-to-day life, suitability of living accommodation, and contribution to society, by setting new directions, such as safeguarding, carer support, and local authorities’ duty to meet older residents’ needs [7]. However, “real-world” outcomes for people with cognitive impairment have not been fully explored because of methodological difficulties. One potential reason is that studies have typically only included individuals who choose to respond to social surveys, whereas people facing adverse circumstances may tend not to respond. Although it is difficult to demonstrate this scientifically, according to Ito [8], who visited non-respondents to a social survey who were 75 years and over, approximately one-third had mild cognitive impairment or dementia and exhibited complex difficulties that were not easily supported.

Gnjidic et al. [9] followed 1705 community-dwelling older men for 5 years and found that 125 (7.3%) men were institutionalized over the study period. The same study reported that mild cognitive impairment was not a predictor of early institutionalization but became a significant predictor after 3.4 years of follow-up. However, the participants were all men, which limited the generalizability of the findings.

Risk factors for institutionalization have been reported when the target population is generalized to all older people, rather than including only those with cognitive impairment. One systematic review [10] identified the following risk factors: older age, low self-rated health status, functional and cognitive impairment, dementia, previous nursing home placement, and a large number of prescriptions. Beswick et al. [11] assessed 89 randomized controlled trials focused on community-based multifactorial interventions to protect community living. They reported that complex interventions, rather than a particular type of intervention, helped older people to continue living at home, and that these interventions could be tailored to meet individual needs and preferences. In Japan, Kazuya [12] reported that informal care was central to community living for older people with disability using long-term care. Similarly, Ohwaki [13] found that having friends was an important predictor of continued home care and potentially prevented institutionalization for older people. However, to the best of our knowledge, the risk factors for older people with cognitive impairment to discontinue community living have not been fully explored. Understanding this issue is essential for building age-friendly communities.

Currently, 28.4% of the total population in Japan is over 65 years old, and more people with cognitive impairment continue to live in the community; such individuals account for approximately 33% of people aged 85 years or over according to a study [2]. There is a research gap concerning the experiences of people with cognitive impairment and protective factors to help this vulnerable population to maintain community living. We identified 198 people who scored < 24 on the MMSE in a baseline survey conducted in 2016 that included all community-dwelling people aged 70 years or over in one Tokyo district [14, 15]. The present study aimed to 1) re-access individuals with cognitive impairment from that cohort and explore their current situation; and 2) identify factors associated with all-cause discontinuation of community living.

## Methods

### Methodology

Various methods have been used in Japan to clarify the experiences of community-dwelling older people in the real world. Repeated access to community residents is often obtained using epidemiological surveys. In such surveys, researchers re-access baseline survey respondents. However, it is often difficult to access people who are no longer living in a particular community (i.e., those who have moved to geriatric institutions or died). Attempts to access hospitals or community support centers listed in a baseline survey are often unproductive, and the staff of such institutions typically cannot provide information because of privacy protection. Consequently, conventional social surveys are of limited use in determining real-world outcomes for community-dwelling older people at risk for discontinuation of community living.

To overcome these limitations, we used a community-based participatory research (CBPR) framework [15]. In addition to using conventional mailing and telephone contact to re-access residents, we created a base camp for our research that provided a comfortable place for community residents to spend time, and in which researchers and community workers could collaborate with community residents. The center was open from 11:00 to 16:00 4 days a week and was located in the center of a housing complex district. Anyone could visit the center and enjoy free tea, coffee, and a snack. Three to five staff members, including at least one full-time researcher with a Ph.D., were present at the center during open hours. Although this center welcomed all residents regardless of age or address, participants from the original study were repeatedly informed about the center. In summary, we acted as both community service providers and researchers to build trust with stakeholders. We held monthly case conferences with the comprehensive support centers from the catchment area, which enabled us to gather information about our study participants.

The research team clarified its priorities in acting as both community service providers and researchers. At the center, decision-making prioritized community service provision over research. Team members always acted according to professional ethical standards.

Because this was a CBPR study, the sample size was limited because we had to maintain close relationships with the participants. Our primary focus was determining factors that influenced the continuation (or not) of living in the community. In the analysis, we regarded the outcome of discontinuation of living in the community as one variable (i.e., as all-cause discontinuation, which comprised death, institutionalization, hospitalization, and moving away).

### Japanese context and setting

Because of factors such as the trend towards smaller family size, aging of caregivers in families, and an increasing number of older people living by themselves [16], the environments surrounding older people have changed. Needs for long-term care have increased because of the greater number of older individuals requiring long-term care, and the lengthening of the care period. Long-term care insurance (LTCI) was launched in Japan in 2000. LTCI is a mandatory program that provides benefits for the long-term care of older persons; all persons aged 40 and over contribute by paying a premium that varies according to income; all persons aged 65 and over can access the same benefits regardless of income [17].

Our study was conducted in Takashimadaira, which is located in northwestern metropolitan Tokyo. Takashimadaira contains the largest housing complex district in Japan, which was built during the 1970s. The aging rate (i.e., the percentage of the total population aged ≥65 years, a widely used indicator of aging in Japan) of this area is approximately 40%. This rate is the same as the predicted aging rate for 2055, which is when societal aging is expected to plateau. We chose this area because it is considered to resemble the Japanese society of the future. A public sector organization, Urban Renaissance Agency (UR) [18], currently manages the housing complex. UR is a semi-governmental organization, originally established in 1955 as Japan Housing Corporation, to address urban and housing issues in Japan. UR is the largest landlord in Japan, currently managing around 2,000,000 residences. Because new residents are not required to have a guarantor (guarantors are customary in Japanese business) many new residents are older people who do not have relatives on whom they can rely for financial support.

### Participants

**Fig. 1:**
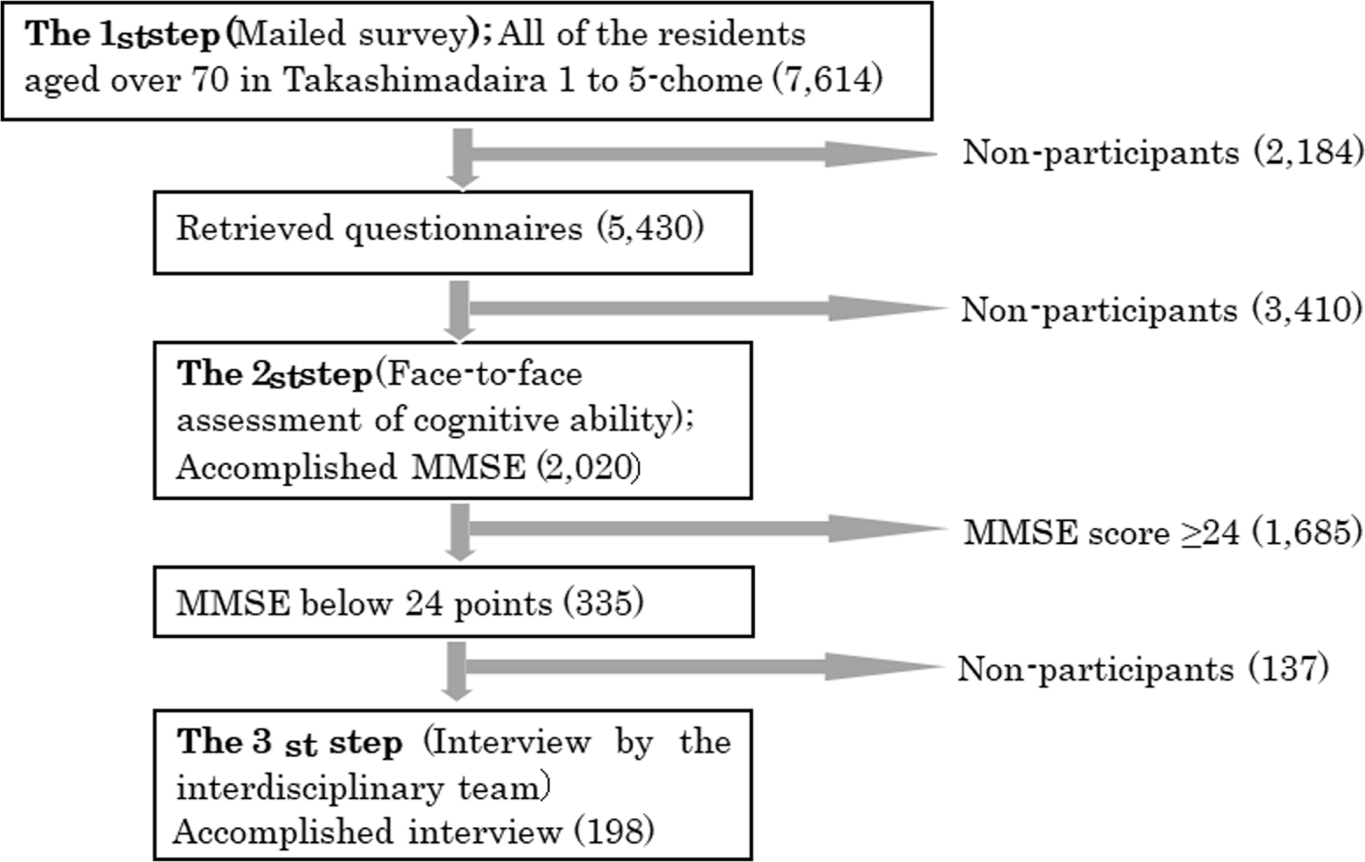
Participant selection flow

Before the study, we conducted a three-step survey of all community residents who were aged 70 years or over in 2016. Figure 1 shows the participant selection flow. Briefly, in the first step, questionnaires were sent to all residents aged 70 and over. In the second step, those who were willing to participate in a face-to-face survey were invited to the surveys in the community center. This assessment included the MMSE. Those with an MMSE score < 24, which is a commonly used cutoff criterion, were potential participants in the subsequent survey. In the third step, a research team that comprised a certified psychiatrist and a gerontologist or a public health nurse made home visits to 198 participants with cognitive impairment who consented to home visits. The CBPR center was opened just after these surveys ended in 2016.

In 2019, 3 years after the baseline survey, we re-accessed these 198 people with cognitive impairment (80 men and 118 women).

### Data collection

To ensure we did not disturb older residents’ community living, we used a stepwise re-access method. First, we mailed letters alerting residents that we would contact them by telephone in a few days. Then, staff with experience in epidemiological research with older people (C.U., a female tenured research fellow with a PhD) telephoned participants to inquire about their current situation. In addition, 62 participants had been helped by our community center and had existing records. Therefore, community center staff who had built face-to-face relationships with residents also telephoned these participants to reassure them that the telephone call was from the research institute and was not a telephone scam. Information about participants who could not be accessed via mail or telephone was obtained with the help of monthly case conferences in the community comprehensive support centers. Permission to re-access participants in a future survey had been obtained during the baseline survey.

### Measurement

The main outcome was participants’ current status (i.e., community living, moved to another community, hospitalized, institutionalized, deceased, or unknown) in 2019.

#### Covariates

All of the following factors were examined in the baseline survey in 2016:

1. Sociodemographic variables.

We used questionnaires to obtain information about participants’ sociodemographic characteristics. Information about long-term care insurance for each participant was obtained from the government.

(2) Cognitive assessment.

MMSE [19, 20] was used to assess participants’ cognitive ability.

(3) Psychological assessment.

Depressive symptoms were assessed using the 15-item Geriatric Depression Scale (GDS-15) [21]. Scores above 5 were considered to indicate the presence of depressive symptoms. Participants’ mental well-being was assessed using the simplified Japanese version of the World Health Organization (WHO)-Five Well-being Index (S-WHO-5-J) [22].

(4) Physical health-related assessment.

Self-perceived health was measured using a single item (see supplementary file).

Frailty was assessed using the Kihon Checklist (KCL) [23], developed by the Japanese Ministry of Health, Labour and Welfare. According to Satake et al. [24], total KCL scores were correlated with frailty, which is defined in the Cardiovascular Health Study criteria. In the current study, total KCL score cutoffs of 7/8 and 3/4 were used to identify frailty and potential frailty, respectively.

(5) Sociological variables.

(i) Relationship with the community.

As described in the report by Ura et al. [14], lack of community participation was defined by participants not attending any of eight social groups. Similarly, trust among neighbors was assessed using a single item (see supplementary file).

(ii) Socioeconomic status.

Perceived current socioeconomic status was assessed using a single item, on a five-point Likert-type scale. Individuals who responded “somewhat poor” and “poor” were classified as having financial disadvantage (see supplementary file). In addition, we asked participants to report their annual income range; those who reported an annual income of < 1,000,000 yen (equivalent to 9200 USD at a currency rate of 108.12 yen/dollar) were classified as having a low income. We used this threshold for the following six reasons: 1) Information about the area median income for older people was not available; 2) The average disposable income for older people in Japan is reported to be 2,100,000 yen [1]; 3) There is no widely used definition of “low-income” in Japan; 4) According to The Department of Housing and Community Development (HCD) [25], 50% of the area median income is the threshold for very-low income; 5) For practical reasons, we were only able to ask about income with rough thresholds, namely, 1,000,000 yen, 3,000,000 yen, 7,000,000 yen, and 10,000,000 yen;

(6) Dementia diagnosis judged by geriatric psychiatric specialists at participants’ homes.

The visiting geriatric psychiatric specialist diagnosed participants using the Diagnostic and Statistical Manual of Mental Disorders, 5th Edition (DSM-5) [26], which was finalized in an interdisciplinary research conference that included more than two certified psychiatrists. The Clinical Dementia Rating (CDR) scale was also used, which is a widely used measure [27]. Furthermore, the visiting psychiatrist also clarified whether participants had already received a diagnosis of dementia in a clinical setting.

(7) Need for social support.

As described in a previous report by Ura et al. [14], participants’ need for social support at home was evaluated on nine domains: 1) dementia subtype diagnosis, 2) medical check-up for physical conditions, 3) continuous medical care, 4) daily living support, 5) support for family members, 6) housing support, 7) long-term care insurance, 8) financial support, and 9) rights protection (see supplementary file). This evaluation was made by two or more visiting experts.

### Statistical analysis

There are various reasons for discontinuation of community living because every person has a unique background. Because the aim of our CBPR approach was to develop an inclusive community in which older people can continue to live, we compared descriptive characteristics of participants who continued community living with those who discontinued community living for any reason (moved to another community, hospitalized, institutionalized, and deceased were combined as all-cause discontinuation in the main analysis). Participants with unknown outcomes were excluded from the analysis. T-tests were used for continuous variables and chi-square tests were used for nominal variables. Next, we performed a multiple logistic regression analysis that included factors significant in the bivariate analyses (threshold set at a *p*-value < 0.05). To avoid multicollinearity, CDR score was not included in the multiple logistic regression analysis because it was clinically obvious that it correlated with dementia diagnosis. We then confirmed that the variance inflation factor was less than 2 for all the independent variables included in the multiple logistic regression analysis. Continuous variables that did not have a cutoff criterion (i.e., MMSE, S-WHO-5-J) were converted into two-item variables and divided into two groups with cutoff points based on the average score. That is, because the average MMSE score was 20.1, the cutoff point was set at 20/21. Similarly, the average S-WHO-5-J score was 8.8, and the cutoff point was set at 8/9. A *p*-value < 0.05 was regarded as statistically significant.

## Results

### Main outcome

Table 1 shows participant characteristics and outcomes. Three years after the baseline survey, 126 (63.6%) of 198 community-dwelling older people with cognitive impairment who were selected from the epidemiological flow had continued living in the same place. Fifty-eight (29.3%) had discontinued community living (all-cause discontinuation). Of these, 18 (9.1%) had died, 18 (9.1%) were institutionalized, 9 (4.5%) were hospitalized, and 13 (6.6%) had moved out of the community. Because of privacy protection, we could not obtain the details of those that had moved out of the community. For example, we heard that one participant had moved some distance to their hometown, which they had not visited for a long time and in which they had no relatives. We suspect that this participant had moved to an institution in their hometown, but we could not confirm this. The remaining 14 (7.1%) participants dropped out because no information was available.

**Table 1:** Participant characteristics and outcomes

	Continued community living	Discontinued community living				Missing information
		Died	Institutionalized	Hospitalized	Moved out of the community	
N (%) of 198	126 (63.6%)	18 (9.1%)	18 (9.1%)	9 (4.5%)	13 (6.6%)	14 (7.1%)
Age, mean ± SD	80.1 ± 5.2	84.3 ± 7.3	83.1 ± 5.1	80.2 ± 6.6	83.3 ± 7.7	81.2 ± 7.8
Educational years, mean ± SD^a^	10.7 ± 2.6	12.5 ± 3.1	11.6 ± 3.0	11.8 ± 2.6	9.5 ± 1.7	11.4 ± 2.6
MMSE score, mean ± SD	20.5 ± 2.8	19.2 ± 4.8	18.9 ± 5.0	20.2 ± 2.4	19.1 ± 4.0	19.1 ± 5.7
N (%) males	49 (38.9)	11 (61.1)	7 (38.9)	3 (33.3)	3 (23.1)	7 (50.0)
N (%) living alone	55 (43.7)	4 (22.2)	9 (50.0)	4 (44.4)	9 (69.2)	9 (64.3)
N (%) married^b^	59 (47.2)	11 (64.7)	8 (44.4)	3 (37.5)	2 (16.7)	4 (28.6)
N (%) certified long-term care insurance	24 (19.0)	10 (55.6)	10 (55.6)	3 (33.3)	6 (46.2)	6 (42.9)

^a^Number of missing cases = 8. ^b^Number of missing cases = 4

*MMSE* Mini-Mental State Examination

### Factors associated with discontinuation of community living

The results of the bivariate analyses are shown in Table 2. Factors associated with discontinuation of community living were being older, LTCI certification, lower MMSE scores (poor cognitive ability), lower S-WHO-5-J scores (poor well-being), poor self-perceived health, frailty, lack of trust of neighbors, DSM-5 dementia diagnosis, higher CDR score (severe stage for clinical rating of dementia), unmet housing support needs, and unmet rights protection needs.

**Table 2:** Comparison of participants who continued community living and those who did not

			Continued community living (*N* = 126)	Discontinued community living (*N* = 58)	χ^2^	F value	Missing cases
(1) Sociodemographic variables							
Age, mean ± SD			80.1 ± 5.2	83.1 ± 6.6		F(1,182) = 10.96, *p* = 0.001	0
Sex		Male	49 (67.1)	24 (32.9)	χ^2^ (1, *N* = 184) = 0.10, *p* = 0.748		0
		Female	77 (69.4)	34 (30.6)			
Educational years, mean ± SD			10.7 ± 2.6	11.4 ± 2.9		F(1,175) = 2.55, *p* = 0.112	7
Type of household		Living alone	55 (67.9)	26 (32.1)	χ^2^ (1, *N* = 184) = 0.02, *p* = 0.881		0
		With others	71 (68.9)	32 (31.1)			
Married		Yes	59 (71.1)	24 (28.9)	χ^2^ (1, *N* = 180) = 0.20, *p* = 0.659		4
		No	66 (68.0)	31 (32.0)			
Long-term care insurance		Certified	24 (45.3)	29 (54.7)	χ^2^ (1, *N* = 184) = 18.56, *p* = 0.000		0
		Not certified	102 (77.9)	29 (22.1)			
(2) Cognitive assessment							
MMSE score, mean ± SD			20.5 ± 2.8	19.2 ± 4.3		F(1,182) = 5.73, *p* = 0.018	0
(3) Psychological assessment							
Depressive symptoms (GDS-15)		High risk (≥5)	62 (65.3)	33 (34.7)	χ^2^ (1, *N* = 175) = 1.06, *p* = 0.304		9
		No high risk (< 5)	58 (72.5)	22 (27.5)			
Mental well-being (S-WHO-5-J), mean ± SD			9.2 ± 3.5	7.8 ± 3.9		F(1,177) = 6.23, *p* = 0.013	5
(4) Physical health-related assessment							
Self-perceived health		Good	99 (73.9)	35 (26.1)	χ^2^ (1, *N* = 181) = 6.90, *p* = 0.009		3
		Not good	25 (53.2)	22 (46.8)			
Frailty		Healthy (0–3)	49 (81.7)	11 (18.3)	χ^2^ (2, *N* = 184) = 11.29, *p* = 0.004		0
		Pre-frailty (4–7)	40 (71.4)	16 (28.6)			
		Frailty (8–25)	37 (54.4)	31 (45.6)			
(5) Sociological variables							
(i) Relationship with the community							
Lack of social participation		Yes	41 (70.7)	17 (29.3)	χ^2^ (1, *N* = 158) = 0.03, *p* = 0.860		26
		No	72 (72.0)	28 (28.0)			
Lack of trust in neighbors		Yes	8 (40.0)	12 (60.0)	χ^2^ (1, *N* = 170) = 8.34, *p* = 0.004		14
		No	108 (72.0)	42 (28.0)			
(ii) Socioeconomic status							
Having a financial disadvantage		Yes	43 (72.9)	16 (27.1)	χ^2^ (1, *N* = 176) = 0.71, *p* = 0.401		8
		No	78 (66.7)	39 (33.3)			
Annual income		Less than one million yen	25 (80.6)	6 (19.4)	χ^2^ (1, *N* = 170) = 2.93, *p* = 0.087		14
		One million yen or more	90 (64.7)	49 (35.3)			
(6) Dementia diagnosis							
DSM-5 diagnosis		No Dementia	86 (76.8)	26 (23.2)	χ^2^ (1, *N* = 184) = 9.15, *p* = 0.002		0
		Dementia	40 (55.6)	32 (44.4)			
Clinical Dementia Rating assessment		Normal (0)	59 (81.9)	13 (18.1)	χ^2^ (2, *N* = 184) = 27.28, *p* = 0.000		0
		Mild (0.5 + 1)	65 (67.0)	32 (33.0)			
		Not mild (2 + 3)	2 (13.3)	13 (86.7)			
(7) Social support needs							
Total needs		0	64 (72.7)	24 (27.3)	χ^2^ (2, *N* = 184) = 1.82, *p* = 0.403		0
		1	24 (68.6)	11 (31.4)			
		2+	38 (62.3)	23 (37.7)			
Dementia subtype diagnosis		Yes	37 (64.9)	20 (35.1)	χ^2^ (1, *N* = 184) = 0.49, *p* = 0.485		
		No	89 (70.1)	38 (29.9)			
Medical check-up for physical conditions		Yes	9 (60.0)	6 (40.0)	χ^2^ (1, *N* = 184) = 0.54, *p* = 0.461		0
		No	117 (69.2)	52 (30.8)			
Continuous medical care		Yes	9 (52.9)	8 (47.1)	χ^2^ (1, *N* = 184) = 2.10, *p* = 0.148		0
		No	117 (70.1)	50 (29.9)			
Daily living support		Yes	22 (56.4)	17 (43.6)	χ^2^ (1, *N* = 184) = 3.34, *p* = 0.068		0
		No	104 (71.7)	41 (28.3)			
Support for family members		Yes	21 (56.8)	16 (43.2)	χ^2^ (1, *N* = 184) = 2.95, *p* = 0.086		0
		No	105 (71.4)	42 (28.6)			
Housing support		Yes	2 (25.0)	6 (75.0)	χ^2^ (1, *N* = 184) = 7.32, *p* = 0.007		0
		No	124 (70.5)	52 (29.5)			
Long-term care insurance		Yes	32 (71.1)	13 (28.9)	χ^2^ (1, *N* = 184) = 0.19, *p* = 0.662		0
		No	94 (67.6)	45 (32.4)			
Financial support		Yes	4 (50.0)	4 (50.0)	χ^2^ (1, *N* = 184) = 1.32, *p* = 0.250		0
		No	122 (69.3)	54 (30.7)			
Rights protection		Yes	8 (44.4)	10 (55.6)	χ^2^ (1, *N* = 184) = 5.34, *p* = 0.021		0
		No	118 (71.1)	48 (28.9)			

The values given in parentheses indicate the percentage of cases (n(%))

*MMSE* Mini-Mental State Examination

*GDS-15* The 15-item Geriatric Depression Scale

*S-WHO-5-J* Simplified Japanese version of the World Health Organization (WHO)-Five Well-being Index

*DSM-5* Diagnostic and Statistical Manual of Mental Disorders, Fifth Edition

Next, we performed a multiple logistic regression analysis that included factors that were significant in the bivariate analyses (*p*-values < 0.05). CDR score was excluded, as explained in the Statistical analysis section. The factors associated with discontinuation of community living were LTCI certification, needing housing support, and needing rights protection (Table 3).

**Table 3:** Factors associated with discontinuation of community living: simultaneous multiple logistic regression analysis

	Odds ratio	95% confidence interval	*P*-value
Age (≥80 years)	1.67	0.73–3.84	0.225
Long-term care insurance (Certified)	3.59	1.51–8.53	0.004
MMSE (Low score)	0.81	0.32–2.03	0.650
DSM-5 diagnosis (Dementia)	1.45	0.58–3.61	0.428
S-WHO-5-J (Low score)	1.64	0.73–3.64	0.229
Self-perceived health (Not good)	1.74	0.63–4.79	0.286
Frailty (Frailty)	0.91	0.33–2.49	0.847
Lack of trust in neighbors (Yes)	3.36	0.97–11.67	0.056
Need for housing support (Yes)	6.79	1.09–42.25	0.040
Need for rights protection (Yes)	3.53	1.06–11.76	0.040

*N* = 184

*MMSE* Mini-Mental State Examination

*DSM-5* Diagnostic and Statistical Manual of Mental Disorders, Fifth Edition

*S-WHO-5-J* Simplified Japanese version of the World Health Organization (WHO)-Five Well-being Index

## Discussion

In the current study, the risk factors associated with discontinuation of community living, as a key component of “living on the edge,” were certified under LTCI, needing housing support, and needing rights protection.

One previous study [28] investigating the ecological relationship between social capital and the proportion of people with cognitive decline in Tokyo found that the proportion of people with cognitive decline was higher in districts with higher social capital. However, trust in neighbors, which is an important component of social capital, was not significantly correlated with discontinuation of community living in the present study (*p* = 0.056).

The association between LTCI certification and discontinuation of community living indicates that high-risk older people experiencing difficulty in continuing community living were receiving LTCI. However, despite the assistance provided by LTCI, many older people with cognitive impairment were found to be moving out of the community. In addition to LTCI, further social intervention for housing support and rights protection is needed for older people to continue living in the community.

Our findings suggest that the implementation of housing support and rights protection is important in developing communities in which older people can continue to live despite having cognitive impairment or living alone. Regarding housing support, it is gradually becoming more difficult for older people with cognitive decline and physical frailty to continue living in the same environment. Thus, the gray area between mainstream housing and housing with care will be critical for age-friendly communities. In the UK, specialist housing refers to housing for older people that facilitates independent living but incorporates different levels of care and support. However, according to Harding et al. [29], a financially challenging climate has resulted in a shortage of supply in the private sector, as well as a preference for people with lower levels of physical and health needs. In Japan, housing for older people is now largely divided into independent living in the community (although LTCI is available) and institutional living. Clarifying the care needs of older people to maintain community living is critical to bridge these areas. Unfortunately, we did not have sufficient data to explore this question in more depth in the current study. A further qualitative investigation focused on older people who continue to live in the community and people who move to geriatric facilities is necessary to reveal the housing needs of a variety of older people in the Japanese context.

Regarding rights protection, the presence of a social network among residents, which is another component of social capital, is beneficial. However, social networks are weakening. According to a Japanese Cabinet office poll [30], 35% of citizens do not have a relationship with their neighbors. To build age-friendly communities in which people with cognitive impairment are supported by multiple social networks, the participation of community residents is essential. To achieve this aim, CBPR is effective for revealing the mindsets of various stakeholders in a specific context, which is an important future challenge.

Dementia [31], physical frailty [32], well-being [33], and living alone [34] (which are the main targets of conventional medical intervention) were not correlated with discontinuation of community living in the multivariate analysis. However, LTCI certification, needing housing support, and needing rights protection remained significant factors for discontinuation of community living. In 1961, Japan implemented Universal Health Coverage (UHC), under which Japanese citizens are enrolled in at least one public health insurance scheme. Under the UHC, citizens are free to choose their own healthcare providers as well as their frequency of treatment, regardless of their socioeconomic status. Social needs for healthcare are currently changing, and chronic illnesses, rather than acute illnesses, constitute the major burden for healthcare worldwide [35]. The current study also suggested that medical conditions did not determine continuation of community living. Our results suggest that an important goal for geriatric medicine is to bridge medical care and social support, such as long-term care, housing support, and rights protection, for older people “living on the edge.”

Our study had some limitations. First, we did not use brain imaging or blood tests to rule out intracranial lesions or other physical conditions that lead to cognitive decline. Second, the study was limited to one district in Tokyo. Third, we were only able to obtain approximate income data. Finally, our results may have been affected by self-selection bias.

## Conclusions

Three years after the baseline survey, 29.3% of people with cognitive impairment had discontinued community living. Despite having cognitive impairment or living alone, older people could continue to live in the community if their needs for housing support and rights protection were fully met. Both social interventions and medical interventions are important for the development of age-friendly communities.

## Supplementary Information

**Additional file 1.**

## Data Availability

The datasets used and/or analyzed during the current study are available from the corresponding author on reasonable request.

## Abbreviations

MMSE: Mini-Mental State Examination
CBPR: Community-based participatory research
GDS-15: The 15-item Geriatric Depression Scale
S-WHO-5-J: Simplified Japanese version of the World Health Organization (WHO)-Five Well-being Index
KCL: Kihon Checklist
DSM-5: Diagnostic and Statistical Manual of Mental Disorders, 5th Edition
CDR: Clinical Dementia Rating
