# Supplementary material for: Living on the edge of the community: factors associated with discontinuation of community living among people with cognitive impairment
Source: BMC Geriatr. 2021 Feb 19;21:131. doi: 10.1186/s12877-021-02084-2 (PMC7893898; doi:10.1186/s12877-021-02084-2)
Supplement: Supplementary file 1 — Additional file 1. [file 12877_2021_2084_MOESM1_ESM.docx]

Supplementary file

Measurement

(4) Physical health-related assessment

Questions concerning “Self-perceived health”

In general, how would you rate your health?

1) Very good; 2) Good; 3) Fair; 4) Poor.

*Answers of “very good” or “good” were recoded as “good”, and other answers as “not good.”

(5) Sociological variables

(i) Questions concerning “Relationship with the community”

(i)-1: Do you attend the following social groups?

1) Neighborhood associations (yes/no)

2) Social clubs (yes/no)

3) Sports clubs (yes/no)

4) Volunteer clubs (yes/no)

5) Senior clubs (yes/no)

6) Alumni associations (yes/no)

7) Occupational associations (yes/no)

8) Other social groups (yes/no)

*Individuals who answered “no” to all items were classified as “lack of social participation”.

(i)-2: Do you trust your neighbors?

1) Strongly disagree; 2) Disagree; 3) Neither agree nor disagree; 4) Agree; 5) Strongly agree.

*Responses of “disagree” or “strongly disagree” were classified as indicating “lack of trust in neighbors”.

(ii) Questions concerning “Perceived current socioeconomic status”

How would you rate your current socioeconomic status?

1) Affluent; 2) Somewhat affluent; 3) Neither affluent nor poor; 4) Somewhat poor; 5) Poor.

*Individuals who answered “somewhat poor” and “poor” were classified as “having financial disadvantage”.

(7) Need for social support

Questions concerning “Need for social support”

1. Does the participant need dementia subtype diagnosis?
2. Does the participant need medical check-up for physical conditions?
3. Does the participant need continuous medical care?
4. Does the participant need daily living support?
5. Does the participant need support for family members?
6. Does the participant need housing support?
7. Does the participant need long-term care insurance?
8. Does the participant need financial support?
9. Does the participant need rights protection?

*These items were evaluated by the visiting geriatric psychiatric specialist at home.
